# A Novel Real-Time Autonomous Crack Inspection System Based on Unmanned Aerial Vehicles

**DOI:** 10.3390/s23073418

**Published:** 2023-03-24

**Authors:** Kwai-Wa Tse, Rendong Pi, Yuxiang Sun, Chih-Yung Wen, Yurong Feng

**Affiliations:** 1 Department of Aeronautical and Aviation Engineering, The Hong Kong Polytechnic University, Kowloon 999077, Hong Kong; kwai-wa.tse@connect.polyu.hk (K.-W.T.);; 2 Department of Mechanical Engineering, The Hong Kong Polytechnic University, Kowloon 999077, Hong Kong

**Keywords:** crack detection, crack localization, autonomous inspection, YOLOv4, attention module, deep learning, unmanned aerial vehicles, UAS

## Abstract

Traditional methods on crack inspection for large infrastructures require a number of structural health inspection devices and instruments. They usually use the signal changes caused by physical deformations from cracks to detect the cracks, which is time-consuming and cost-ineffective. In this work, we propose a novel real-time crack inspection system based on unmanned aerial vehicles for real-world applications. The proposed system successfully detects and classifies various types of cracks. It can accurately find the crack positions in the world coordinate system. Our detector is based on an improved YOLOv4 with an attention module, which produces 90.02% mean average precision (*mAP*) and outperforms the YOLOv4-original by 5.23% in terms of *mAP*. The proposed system is low-cost and lightweight. Moreover, it is not restricted by navigation trajectories. The experimental results demonstrate the robustness and effectiveness of our system in real-world crack inspection tasks.

## 1. Introduction

Monitoring the structural health of civil infrastructures is crucial to society from the perspective of public safety. Inevitably, infrastructures deteriorate by human activities and natural erosion over time. Concrete cracks, steel corrosion, bolt loosening, and delamination are typical structural distresses. Structure health monitoring and inspection tasks are essential to detect these distresses early and reduce the potential disaster risks in time. Traditionally, crack inspection tasks have been performed by certificated engineers. It usually takes weeks or even months to complete a large-scale and complex infrastructure inspection, whereas the inspection report varies from the judgment of different inspectors. Besides the traditional methods, recent civil engineering practitioners rely on inspection devices with remote sensing methods to obtain construction health information. For example, cracks can be found by assessing the features of the ultrasonic signals [1], and optical fibers have been used in monitoring cracks [2]. These sensing techniques can feasibly achieve real-time performance, but they always require extensive devices and supporting facilities to perform the inspection task. From these perspectives, the traditional inspection tasks are time-consuming, subjective, cost-ineffective, and labor-intensive. This work aims to provide a fully onboard UAV inspection system to perform crack inspection and localization, which can effectively locate the crack positions for real-world application.

Apart from the above-mentioned techniques, in the past decade, a wealth of research has emerged to provide image-processing-based methods to examine cracks without additional sensors [3,4]. Many methods are proposed based on deep learning and impressive results have been demonstrated [5,6,7,8,9,10]. However, those methods heavily rely on the computing capability of desktop computers, so it is not feasible to perform real-time inspection onboard, which is essential for real-world industrial inspections.

With the recent advancement of unmanned aerial vehicles (UAV) [11], visual inspection systems can be deployed fully onboard to perform crack detection and localization tasks. Nooralishahi et al. [12] presented various case studies that demonstrated the effectiveness of employing drones to facilitate the inspection of hard-to-reach areas while the work reported in [13] explored the time and applicability of using UAV for building inspection activities, showing that UAVs have great potential to improve efficiency and effectiveness of building maintenance and management. The small UAV is able to fly across large-scale complex structures to identify and localize the cracks. Kim et al. [14] presented a crack identification strategy that combined the aerial images acquired from a UAV and the information from the ultrasonic displacement sensor to estimate the length and width of the crack. Moreover, Li et al. [15] extracted the crack information using four laser emitters for crack image acquisition. The laser-obtained images were geometrically adjusted using the four-point linear correction algorithm. Apart from the perspectives of crack information extraction, Yu et al. [16] developed a fast feature-based stitching algorithm to detect cracks on the large panorama using an off-the-shelf DJI UAV for concrete bridge monitoring. Towards localizing cracks in concrete structures using a UAV, Woo et al. [17] proposed a method utilizing relative positions between reference objects in UAV-captured images and revealed errors in the range of 24–84 mm and 8–48 mm on the x- and y- directions. In their study, the size of the reference object was first estimated by a point-cloud-based method, and the unit pixel size was then obtained to estimate the relative positions of the cracks using the point-cloud technique, image stitching, and homography matrix algorithms. Towards developing an instant inspection system, Saleem et al. [18] introduced an instant bridge visual inspection method using a UAV by an image capturing and geo-tagging system (ICGT) and a deep convolution neural network approach. ICGT controls the camera shutter and paired each captured image with its responding IMU, LiDAR, and GPS data with synchronized timestamps. Kim et al. [19] presented an automatic damage detection and bridge condition evaluation by constructing a point-cloud-based 3D modeling for the target bridge. Many studies have demonstrated crack detection using UAVs. However, most of them are still not fully working onboard or are cost-ineffective. Therefore, a lightweight and fast crack detector that can be deployed onboard is desired.

Redmon et al. [20] designed the first version of YOLO in 2015 [20]. In 2016, YOLOv2 [21] was proposed to improve the accuracy of the bounding box. After that, YOLOv3 [22] was developed by employing multi-scale prediction. There were more predicted bounding boxes than in the former versions. In addition, YOLOv4 [23] was then proposed by supplementing three main features: (1) using multi-anchors for single ground truth, (2) eliminating grid sensitivity, (3) adding complete-intersection over union (CIoU) loss. Recently, a new model termed YOLOv5 [24], possessing higher object detection capability, was developed. YOLOv5 employed Mosaic to conduct data augmentation rather than CutMix [25]. Several novel modules extracting features deeply were added into the backbone. Moreover, a novel loss function termed Generalized IoU [26] was employed in YOLOv5. Among various YOLO models, YOLOv4 is one of modern object detectors offers previse detection performance and high frame rate per second (FPS), making it popular to be applied on UAVs to execute real-time inspection tasks. YOLOv4 is easy to annotate and train. It has a prominent accuracy on object classification task. Moreover, considering the limitation of UAV computing capability and UAV energy limitation, YOLOv4 achieves fast inference speed on Jetson TX2, which is ideal for edge computing for inspection tasks. Additionally, YOLOv4 is designed to be scalable and flexible for different use cases, developers only need to change the network configuration and weights file if the object of interest for inspection is changed. Moreover, it is feasible to make updates to YOLOv4 with new features and improvements. Since original YOLO models do not perform well in detecting tiny objects. To address this problem, attention mechanisms are introduced to enhance the model performance on small objects in this work.

An attention mechanism is the approach that can automatically focus a network on some areas to capture the features. In this section, four typical attention modules are reviewed. SE-Net [27] is a kind of channel-based attention module, which employs a fully-connected layer to extract channel-related features. Based on SE-Net, ECA-Net [28] replaces the fully-connected layer with a one-dimensional convolution layer to decrease the number of parameters. The convolutional block attention module (CBAM) [29] has simultaneously focused on the features derived from spatial and channel spaces. Furthermore, Hou et al. [30] introduced the location information to the channel-based features. This method feasibly increases the network’s receptive field without increasing excessive parameters. Since the attention mechanism helps the model to capture the features of tiny objects, Yu et al. [31] incorporated the attention mechanism into YOLOv3 for vision-based defect inspection, and Sun et al. [32] introduced an improved YOLOv4 based on the attention mechanism and SqueezeNet for person detection. Indeed, a learning-based crack inspection system with an attention mechanism is a novel approach in the research areas of UAVs.

Several researchers have attempted to conduct autonomous crack inspection using UAVs with a deep learning approach. However, some issues still require in-depth investigation to enhance the inference speed and localization accuracy. To address these issues, in this work, we aim to provide a fast crack inspection system to accurately detect and localize cracks in the world coordinate system. The contributions of this paper are summarized as follows:

1. A deep learning-based crack detection method is proposed. We have built a dataset that contains 4000 crack images with three types of concrete textures. The detector shows promising performance in crack detection on unseen cracks. No prior knowledge of the cracks in the structure is needed.
2. The improved YOLOv4-SE and YOLOv4-tiny-SE incorporating attention mechanism in neural networks are designed. We have proved that our improved models outperformed the YOLOv4-original models with higher *mAP* performance on multiple tests.
3. The fully onboard crack localization system is developed. Our system solely utilizes an RGBD camera and precisely locates the crack positions with cm-level accuracy. Moreover, the autonomous UAV system with two inspection trajectories, straight-line and zig-zag, is designed to perform crack inspection tasks for the structure.
4. We present extensive test results in different experimental setups to validate our system. Our code, dataset, and the pre-trained weights are released as an open-source package to the research community.

The rest of this paper is constructed as follows. We discuss the hardware and software components in Section 2.1. Dataset preparation, inspection path planning, localization techniques, and implementation of improved crack detectors with attention mechanisms are presented in Section 2.2, Section 2.3 and Section 2.4. The experiment results are presented in Section 3. More discussion on the experimental results and the future work are provided in Section 4. Finally, the paper is concluded in Section 5.

## 2. Materials and Methods

### 2.1. System Overview

#### 2.1.1. The UAV Hardware Components

**Figure 1:**
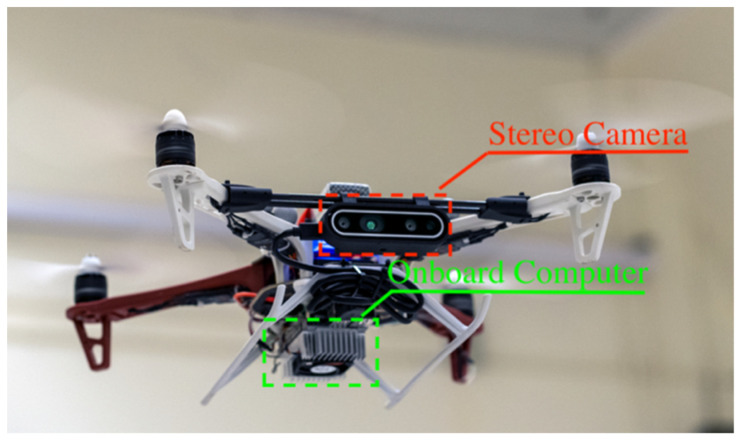
UAV with RGBD stereo camera [33].

As shown in Figure 1, the proposed system utilizes the DJI Flamewheel 450 airframe as the main body of the hardware platform, and utilizes the Pixhawk 4 mini as a flight controller for the navigation system. The aircraft has an onboard computer NVIDIA Jetson TX2 with the Intel RealSense D455 RGBD camera for crack inspection. The deep learning models for crack detection are trained offline in the workstation with the specifications listed in Table 1.

The crack detection models are deployed to the onboard airborne computer NVIDIA Jetson TX2. All the inspection modules work online with satisfied real-time detection performance.

#### 2.1.2. The Inspection System Software Architecture

**Figure 2:**
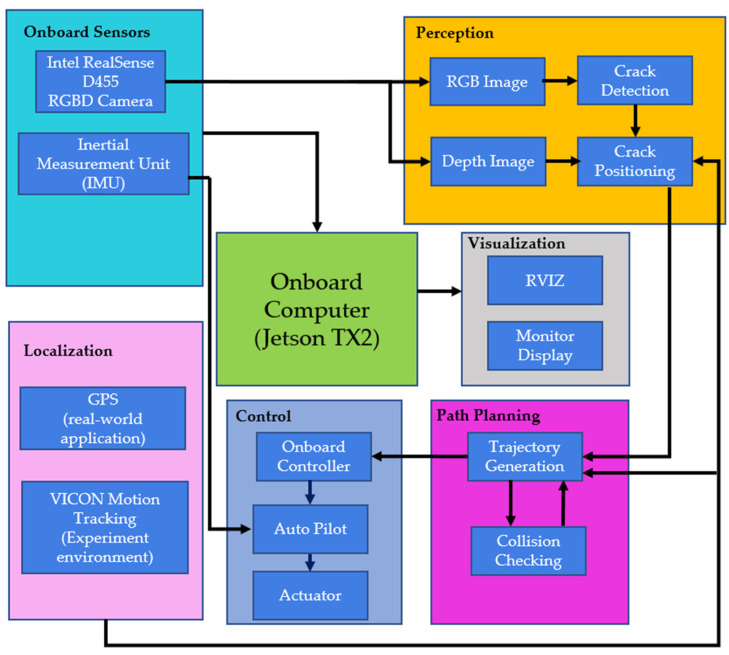
The software architecture of our UAV inspection system.

The aircraft has an onboard computer NVidia Jetson TX2, which processes the system modules that include a perception module, a path planning module, a control module, and a localization module, as shown in Figure 2. The Intel RealSense D455 Depth camera is the only sensor for the proposed system to perceive the environment. The compressed depth-align RGB images are used in the perception module for the detector to identify cracks. The D455 camera calculates the depth values for each pixel. The depth-to-color-align frame, called depth frame in this paper, is then generated and further used to calculate the crack positions in different coordinate systems.

The crack localization method is highly related to the camera and aircraft pose but is independent from the UAV inspection trajectory. The proposed system is verified with various trajectories and achieves promising crack localization results. Thus, our proposed system is not restricted by pre-determined paths, and it is robust for various applications. The aircraft pose acquired from the VICON motion tracking system is used to transform the crack coordinates from the camera frame to the world frame. Moreover, the aircraft pose is one of the critical information for the path planning module. The proposed system is also equipped with a collision-checking technique. The onboard camera Intel RealSense D455 features long-range capabilities with an ideal range from 0.6 m to 6 m and high-depth resolution up to 1280 × 720 pixels at 90 fps. Notably, the depth measurement error of the D455 camera is less than 2% at the range of 4 m. Moreover, the depth sensor field of view (FOV) is 87° × 58° (Horizontal × Vertical). The vertical inspection coverage is around 1.15 m, which is greater than the height of the synthetic banner in our experiments while the inspection distance is set to 1m. The combination of the broader FOV and high-depth resolution enables the inspection system to perform collision checking for the scene.

The crack detector from the former perception module first generates a 2-D bounding box. Then, the region out of the bounding box in the depth frame is calculated. In addition, the collision-checking mechanism of our proposed system utilizes the depth of the crack outer region to determine if it is collision-free for the aircraft in following the trajectories. Moreover, a dynamic path can be generated from the acquired depth information. Moreover, this collision-free active control solely relied on one D455 depth camera was also verified in our previous work [33]. The work presented in this article mainly focuses on crack localization techniques and only covers a small portion of trajectory generation. All modules run in real-timely on the airborne computer, and the performance can be visualized in the 3-D ROS visualization tool (RViz).

### 2.2. Training Dataset Preparation

**Figure 3:**
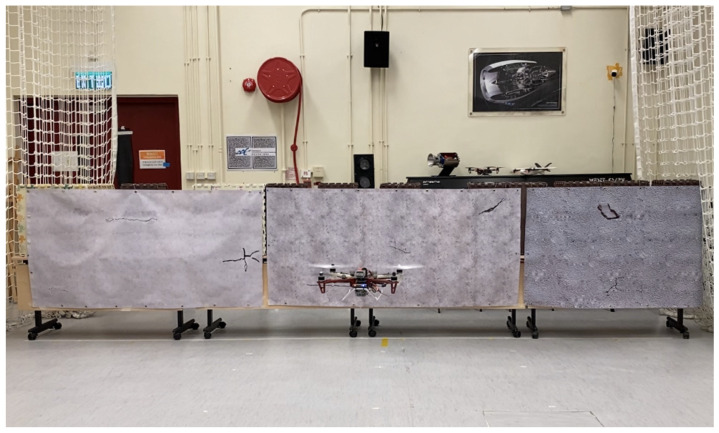
Experimental settings in the laboratory.

**Figure 4:**
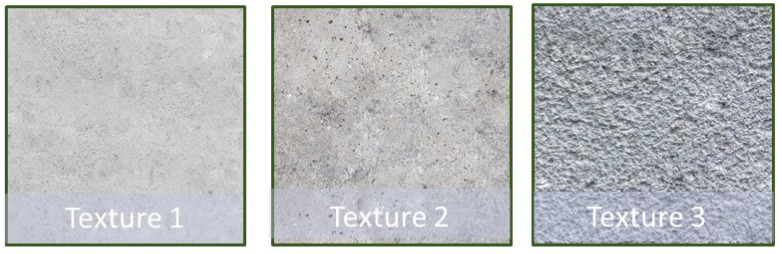
Different concrete textures in the training dataset.

Since we need to conduct many flight tests in this study, an indoor environment in our laboratory is designed to mimic the real-world environment of a part of a long bridge. As shown in Figure 3, the simulated bridge surfaces with different concrete textures and crack patterns are cut from a 6 m × 1 m (width × height) synthetic banner. The three types of concrete textures are chosen (Figure 4) because concrete bridges in the real world are built with different classes of concrete materials and densities with different mechanical properties. They are used to train the detector to recognize different concrete backgrounds as illustrated in Figure 4.

**Figure 5:**
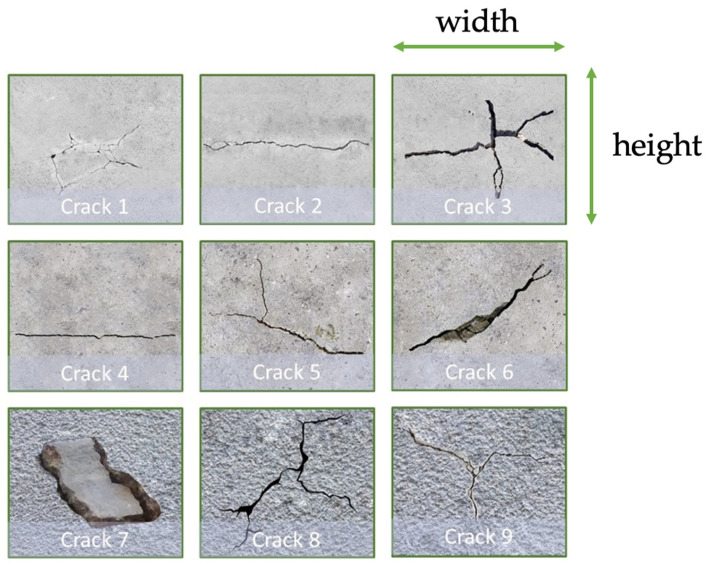
Different types of cracks in the training dataset.

**Figure 6:**
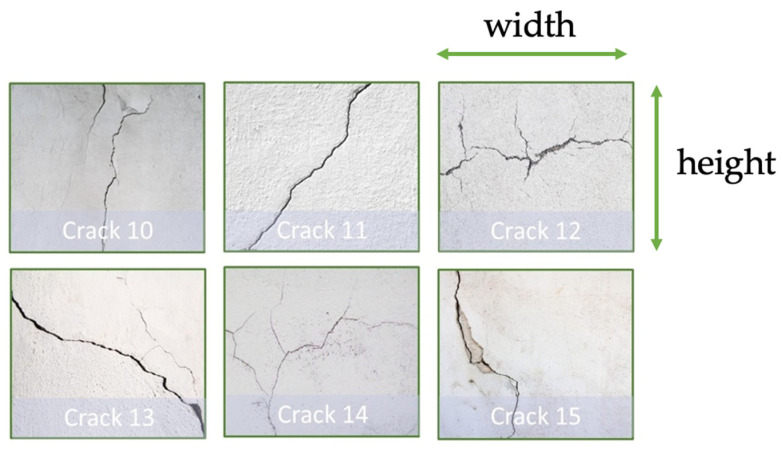
Unseen cracks to test the generalization ability.

Our dataset is acquired by the same onboard RGBD camera, and we label all training images manually. The dataset includes 4000 crack images with 9 types of cracks in different orientations, and the physical dimensions of these cracks are listed in Table 2. The width of the crack is defined as the horizontal dimension of the crack, and the height is defined as the vertical dimension of the crack used in our flight experiments, as illustrated in Figure 5 and Figure 6. Moreover, the thickness of the crack is defined as the size of the gap in the crack defect. In particular, the YOLO detection model preferably requires 2000 different training images for each class or more. Only one class of defect is created in the training images and the onboard detector classifies it if upcoming aerial images contain cracks of this kind and further computes the coordinates of these cracks.

Next, dataset augmentation techniques are incorporated, including image transformation and mosaic augmentation methods, to create additional training images from the existing 4000 image. After data augmentation, the number of training images is 6000. We expect that more training images could increase the detection accuracy and generalization capability on the unseen dataset shown in Figure 6. The physical dimensions of the unseen dataset are depicted in Table 3. For the detection capability, the trained crack detector successfully identifies the crack 1 with the minimum crack thickness of 1 mm, and the crack 7 with the maximum crack thickness of 26 mm in our experiments.

### 2.3. Inspection Path Planning

#### 2.3.1. Path Planning

Path planning aims to find a collision-free path from the starting position to a goal. In the meantime, considering that the battery endurance may not be sufficient to accomplish the entire long-range inspection task, a route with the minimum distance and energy consumption cost is desired.

**Figure 7:**
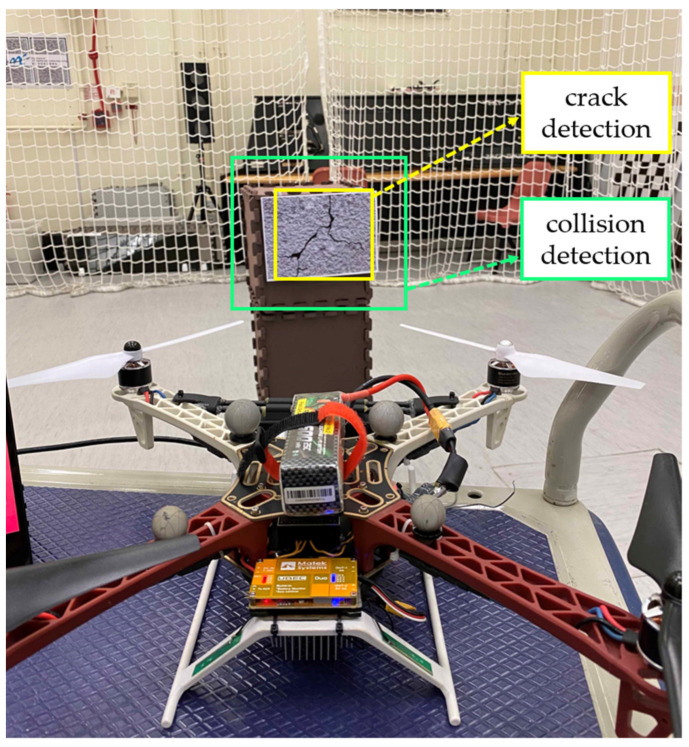
Collision detection using depth information from the surrounding environment.

When constructing a collision-free inspection path in a complex and unknown environment, the proposed inspection system detects the depth information from the outer region once the 2D-bounding box is drawn by the crack detector, as shown in Figure 7. The depth information surrounding the cracks can be captured, and more complicated motion and path planning can be designed using this collision detection technique.

**Figure 8:**
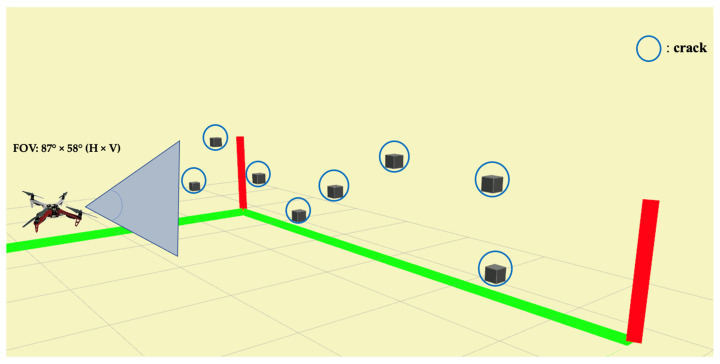
The field of view (FOV) of the inspection window.

**Figure 9:**
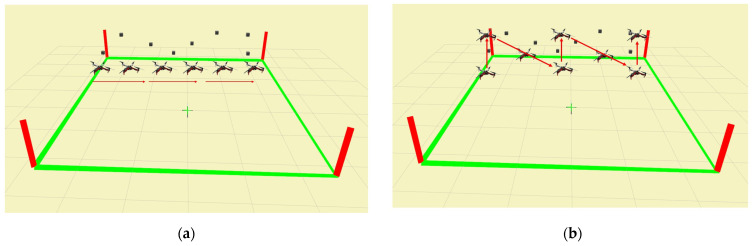
(**a**) The straight-line trajectory; (**b**) The zig-zag trajectory in the experiment.

As mentioned in the Section 2.2, the synthetic banner is designed to mimic the side view of the concreate bridge. Moreover, the inspection task for complex infrastructure is generally well-planned before the task, and the profile and geometric information of the bridge are precisely surveyed. This study does not focus on generating an optimal path for the bridge inspection. As shown in Figure 8, the field of view (FOV) of the RGBD camera is 87° × 58° (Horizontal × Vertical). Two trajectories are pre-determined to examine the crack localization performance. The first trajectory is straight-line, which is the shortest possible route to visit every viewpoint on the synthetic banner, as shown in Figure 9a. Since the vertical coverage is greater than 1m, which is larger than the height of the synthetic banner, the inspection distance between the UAV and the inspection target is set to be 1 m.

To generate a more complex inspection path that maximizes the coverage of the structural surface, another zig-zag trajectory is conducted to demonstrate the crack localization performance of the proposed method. It can be seen in Figure 9b, the zig-zag trajectory is not necessarily designed to follow the exact pattern of the cracks. A zig-zag trajectory efficiently increases inspection coverage. However, the duplicated crack detections are also increased. Consequent to different path planning strategies, an algorithm to reject the duplicated crack detection is introduced, which will be discussed in Section 2.4.3.

#### 2.3.2. Simulation-to-Real

**Figure 10:**
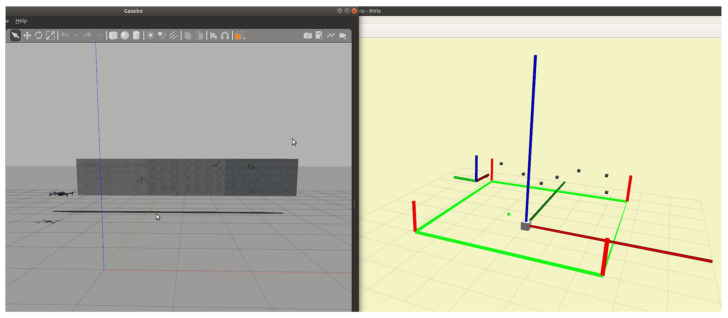
The simulation in Gazebo (**left**) and RViz (**right**) simultaneously.

The proposed inspection system is developed in software-in-the-loop (SIL) mode using the Gazebo simulator and the ROS Visualization tool (RViz) to transfer the simulated experience into the real world. The gazebo is a well-known simulator for robotics research, which features a real-time physics engine with a wide range of UAV sensors and plugins. Before the real flight tests in the laboratory, the motion and path planning components are simulated in Gazebo, and the vehicle motions could be instantly visualized in RViz, as shown in Figure 10.

### 2.4. Crack Detection and Localization

#### 2.4.1. Camera Model

**Figure 11:**
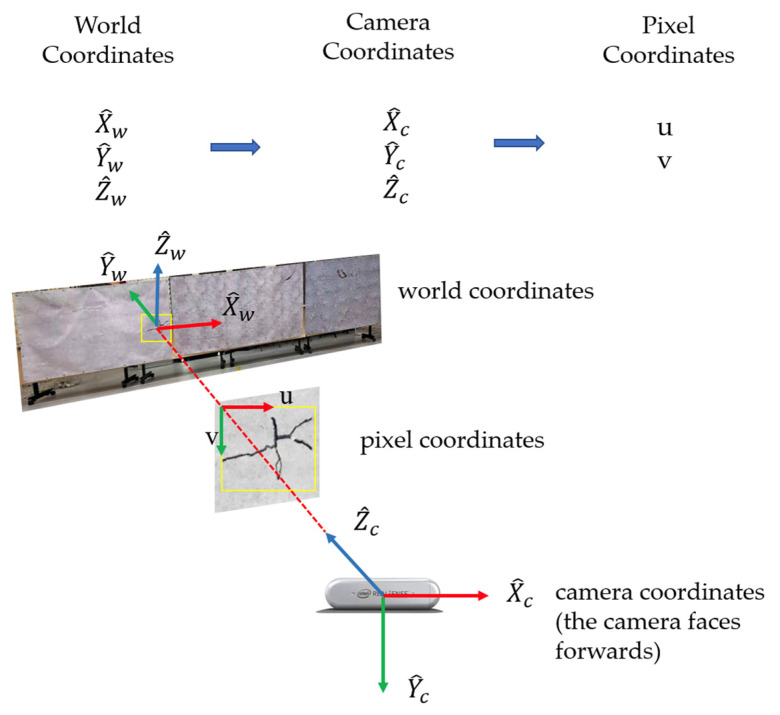
3D-to-2D forward projection.

From the generalized pinhole camera model, the technique to transform a 3D world coordinate point to a 2D pixel coordinate via forward projection is revealed in Figure 11. The 3D world point gets projected into 2D pixel coordinates, which can be mathematically described in Equation (1). α, β, $c_{x}$ , and $c_{y}$  are intrinsic camera parameters, and their values could be retrieved by subscribing to the ROS topic *camera_info*. Moreover, $r_{ij}$  and $t_{x}$, $t_{y}$, $t_{z}$  are extrinsic parameters that address the rotation and translation between the two coordinate systems.

(1) $$z\left[\begin{array}{c} u\\ v\\ 1 \end{array}\right]=\left[\begin{array}{ccc} \alpha & 0 & c_{x}\\ 0 & \beta & c_{y}\\ 0 & 0 & 1 \end{array}\right]\left[\begin{array}{cccc} r_{11} & r_{12} & r_{13} & t_{x}\\ r_{21} & r_{22} & r_{23} & t_{y}\\ r_{31} & r_{32} & r_{33} & t_{z} \end{array}\right]\left[\begin{array}{c} {\hat{X}}_{w}\\ {\hat{Y}}_{w}\\ {\hat{Z}}_{w}\\ 1 \end{array}\right]$$

(2) TCUAV=0010.13−10000−1000001

(3) PUAV=TCCUAVP

(4) PW=TUAVUAVWP

#### 2.4.2. Coordinate Transformation

**Figure 12:**
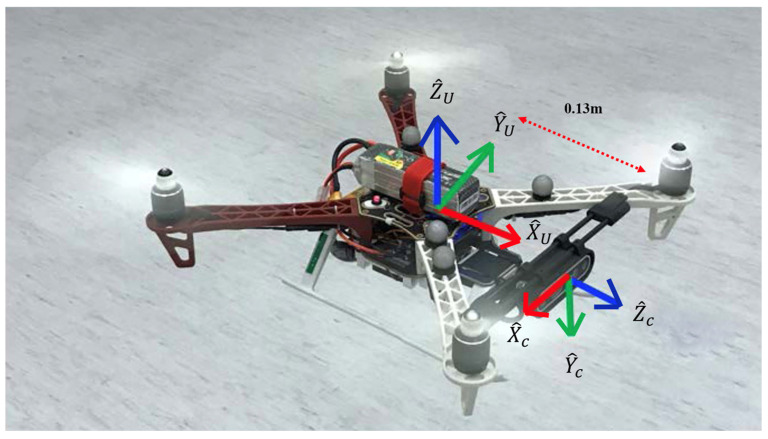
Visualization of camera coordinate and UAV body coordinate.

Upon the generalized pinhole camera model in Equation (1) from the previous section, the problem of crack localization in 3D space can be formulated as a backward 2D-to-3D operation. The camera is moving with the vehicle body, and the vehicle pose relative to the world frame is tracked by the motion capture system (i.e., VICON). TCUAV  in Equation (2) denotes the transformation between the UAV body frame and the camera frame. TCUAV  describes the camera frame is rotated first about y-axis by an angle of +90° and then about z-axis by an angle of −90°. In particular, the translation for the x-axis between two frames is 0.13 m, as shown in Figure 12. At last, the 3D position of the detected crack can be computed by Equations (3) and (4), where PC  and PUAV  represent the 3D positions of the camera and UAV. In addition, TUAVW  denotes the transformation matrix that transforms the body coordinate to 3D world coordinate which is derived from VICON system.

#### 2.4.3. Rejecting Duplicated Crack Detections

**Figure 13:**
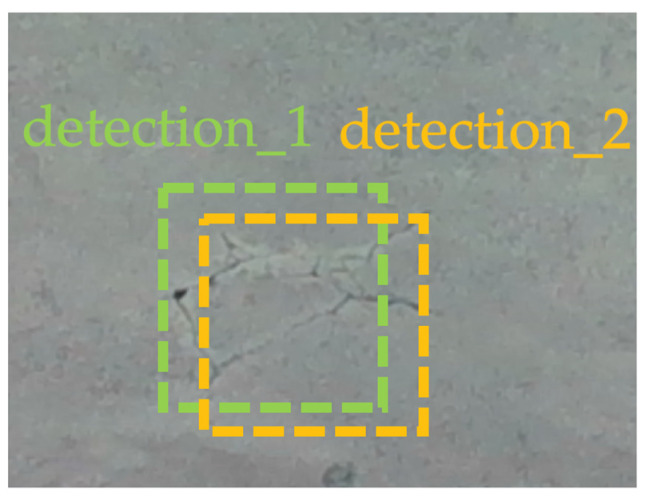
Duplicated detection records on a single crack.

Since the RGB frame rate of the used depth camera is 30 FPS at the resolution of 1280 × 800 and the inference rate of the crack detector model is around 10 FPS, there exists the possibility of overlapped inspection coverage on a single crack, as illustrated in Figure 13. So, a mechanism to reject duplicate detections elaborated in Algorithm 1 is crucial. The distance between two detections can be computed by Equation (5), where *est* denotes the current estimated crack coordinates and *pre* denotes previously identified crack coordinates. Specifically, the distance in 3D space between detection 1 ($x_{1},y_{1},z_{1})$  and detection 2 ($x_{2},y_{2},z_{2}$)  on a single crack can be calculated by Equation (6).

(5) $$Distance=\sqrt{{\left(x_{est}-x_{pre}\right)}^{2}+{\left(y_{est}-y_{pre}\right)}^{2}+{\left(z_{est}-z_{pre}\right)}^{2}}$$

(6) $$Distance\;between\;two\;detections=\sqrt[{}]{{\left(x_{1}-x_{2}\right)}^{2}+{\left(y_{1}-y_{2}\right)}^{2}+{\left(z_{1}-z_{2}\right)}^{2}}$$

However, to deal with the duplicated detection records caused by the overlapped inspection coverage, the distance value between current identified crack and every previous identified crack must be calculated. A distance threshold of 150 mm is chosen in this work. The pseudocodes for rejecting duplicate detection records are stated as follows:
**Algorithm 1**: Rejecting duplicate detection records1:Distance threshold ← 150 mm2:**while** detection function start **do**3:    **if** crack detection flag is **TRUE then**4:        **for** each detected crack **do**5:            calculate the 3D world coordinate of current crack6:            distance difference ← calculate the distance between             current crack and each previous identified crack7:            **if** distance difference < distance threshold **then**8:                 mark it as duplicates detection record9:                 reject duplicate detections10:            
**end if**
11:        
**end for**
12:    
**end if**
13:**end while**

#### 2.4.4. Improved YOLOv4 Models with Attention Mechanism

**Figure 14:**
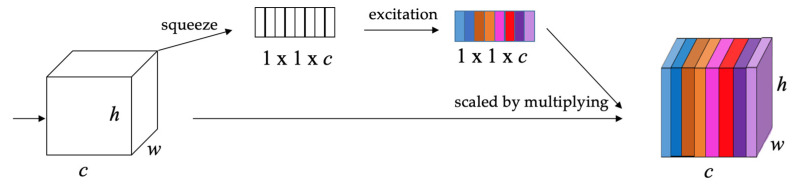
Squeeze-and-Excitation Networks [27].

In this work, we integrate the channel-based attention modules Squeeze-and-Excitation Networks (SENets) [27] into the origin YOLOv4 to enhance the performance of crack detection, as shown in Figure 14. First, the global spatial information is collected in the squeeze module by global average pooling. *h* denotes the height, *w* the width, and *c* the channel of the feature map. Then, the excitation module captures channel-wise relationships and outputs an attention vector using fully connected and non-linear layers. Finally, each channel of the input feature is scaled by multiplying the corresponding element in the attention vector.

**Figure 15:**
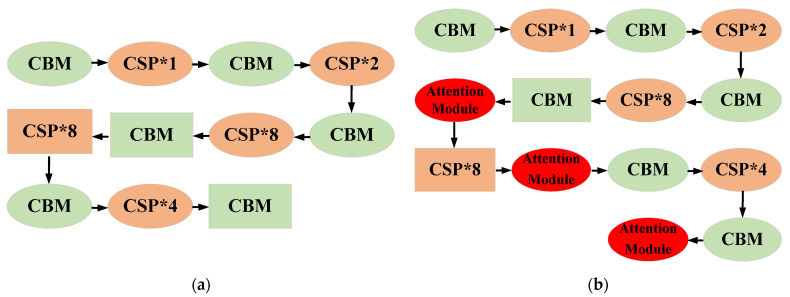
(**a**) The architecture of original YOLOv4; (**b**) improved YOLOv4 with attention modules.

Considering that the backbone is mainly employed to extract image features in YOLO, the attention modules are added to the backbone to improve the detection performance. Both the structure of the origin YOLOv4 and improved YOLOv4 are shown in Figure 15. We can see that these three attention modules are inserted in the three locations of the backbone. The first two locations are in front of the layers whose features are fed into the detection head for feature fusion. The third location is at the end of the backbone of YOLOv4. Adding attention modules into these locations can further extract the features of images. CBM denotes the combinations of Convolutions, Batch normalization, and Mish activation layer. CSP denotes Cross-Stage Partial dense net. The insertion of SENets after the convolutional layers helps the network focus on the most important features of input aerial images, while suppressing less important features. Compared with the concrete surface of the bridge, the crack defective areas are darker. Then, the channel-based attention module captures the difference between the normal surfaces and cracks. YOLOV4-original is employed as the baseline for the performance comparison, our experimental results presented in Section 3 demonstrate that the incorporation of attention module effectively enhances the crack detection performance.

**Figure 16:**
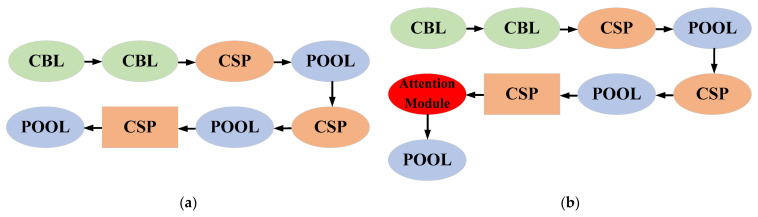
(**a**) The architecture of original YOLOv4-tiny; (**b**) The architecture of improved YOLOv4-tiny with attention module.

In addition, considering the limited computational resource of the on-board computer, a lightweight improved YOLOv4-tiny is also developed to achieve a higher inference rate. The strategy for improving YOLOv4-tiny is the same as the YOLOv4 mentioned earlier with attention modules. It means that the attention module is added to the location whose features is fed into the YOLO Head for detection. The architecture of the original YOLOv4-tiny and improved YOLOv4-tiny are shown in Figure 16. CBL denotes the combination of Convolution, Batch Normalization, and Leaky, respectively.

## 3. Experimental Results

The experiments have been carried out to demonstrate the following aspects of performance of our proposed crack inspection system:

- The crack detection performance of the improved YOLOv4 with attention mechanism on various crack datasets (Section 3.1);
- The real-time multi-cracks detection performance in the flight tests, and the generalization capability on unseen cracks in real flight experiments (Section 3.2);
- The cracks localization performance in real flight experiments with assessments on Root-Mean-Square Errors (RMSE), and crack localization errors in 3D space (Section 3.3).

### 3.1. Crack Detection Performance of the Improved YOLOv4 with Attention Mechanism

To analyze the crack detection performance of our improved YOLOv4 models with the attention mechanism, both the self-collected dataset introduced in Section 2.2 and a public dataset were used for the benchmark. Various models were run offline in the workstation depicted in Table 1. The detection performance comparisons on the customized dataset are listed in Table 4, where the YOLOv4-original and YOLOv4-tiny-original models were treated as baselines for the benchmark. Our YOLOv4-SE and YOLOv4-tiny-SE achieves 90.02% and 85.46% *mAP* on our crack dataset, respectively.

In addition, a public dataset, the UAV Asphalt Pavement Distress (UAPD) dataset [34] is also utilized to evaluate the performance among various YOLOv4 models. In the UAPD dataset, there are 6 classes of cracks but only 3000 crack images, which is insufficient to train robust crack detectors for the 6 classes of cracks. Thus, all trained YOLOv4 models achieved relatively low *mAP* values, as shown in Table 5. Similarly, the YOLOv4-original and YOLOv4-tiny-original models were treated as baselines for the benchmark. Our YOLOv4-SE achieved 48.69% *mAP*, with 3.5% increase over the YOLOv4-original on the UAPD dataset. The performance comparison proves the improved YOLOv4 models not only enhance the crack detection performance on our dataset, but also boost the detection accuracy on other real-world crack dataset with asphalt background.

### 3.2. Multi-Cracks Detection Performance and Generalization Capability

**Figure 17:**
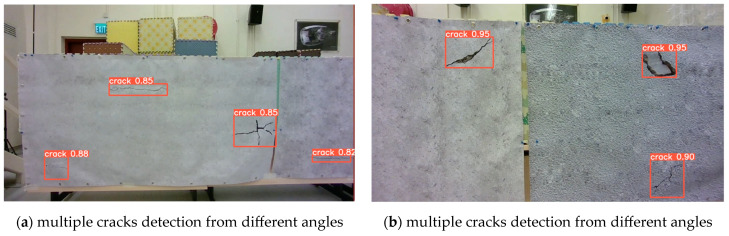
Multiple cracks detection performance in the laboratory environment.

There are numerous cracks in one image streaming from the onboard camera of the UAV. Thus, the capability of detecting multiple cracks at the same time is an important criterion for the crack detector. Figure 17a,b demonstrate the crack detection results of our improved YOLOv4-SE in the laboratory environment.

**Figure 18:**
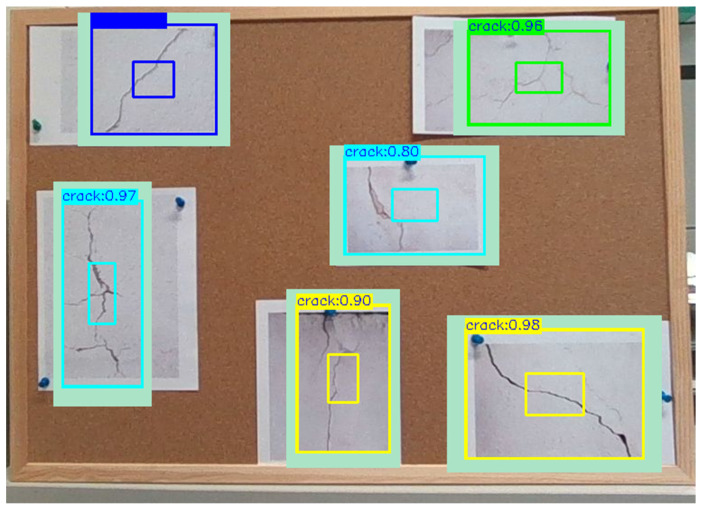
Multiple cracks detection results on unseen crack images.

To verify the generalization capability of our improved YOLOv4-SE, we test some crack images in Figure 18. They were the crack data that our model has never seen before. The results on those unseen data are illustrated in Figure 18.

### 3.3. Cracks Localization Performance

**Figure 19:**
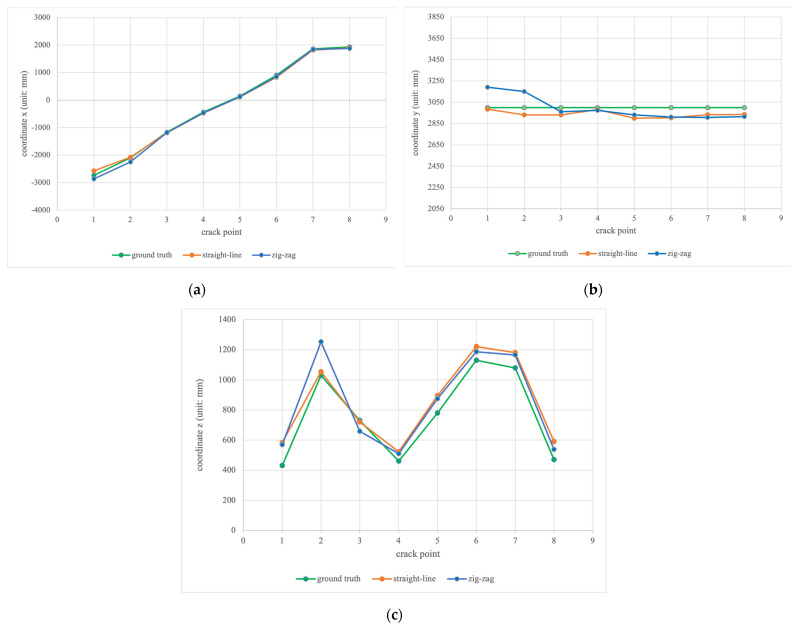
The localization results of (**a**) x coordinates; (**b**) y coordinates; (**c**) z coordinates on the training cracks.

In this work, our improved YOLOv4-SE was deployed on a UAV to detect and localize cracks in the experimental setting displayed in Figure 3. Both training and unseen cracks were used to examine the crack localization performance of the proposed inspection system along two flight trajectories: straight-line and zig-zag trajectories. The positions of the crack are expressed using the center of the crack shape. The ground truth (x, y, z) coordinates of the crack positions are pre-measured. The ground truth positions of the training cracks are presented in upper rows, and the localization measurement/estimation values and errors along the straight-line and zig-zag trajectories are listed in the middle and bottom rows in Table 6, respectively. Notably, Crack 9 is out of the geo-fence of the VICON tracking system in the laboratory. Thus, the experimental data of Crack 9 are excluded in this section. The crack localization results on training cracks are also visualized in Figure 19. The green line plots the ground truth positions, while orange lines and blue lines plot the localization measurements along straight-line and zig-zag inspection trajectories, respectively, as shown in Figure 19. In general, we can observe that the localization measurements of cracks positions follow the ground truth. Particularly, the localization performance from our work achieves the minimum errors of x, y, and z are −11, −16, and −10 mm, respectively, on the training cracks, while the maximum errors of x, y, and z are 170, 190, and 223 mm, respectively. Based on data presented in Figure 19, the localization performance of x coordinate on training cracks is slightly better than the localization performance of y and z coordinates. Details about the experiments can befound in the Supplementary Video.

**Figure 20:**
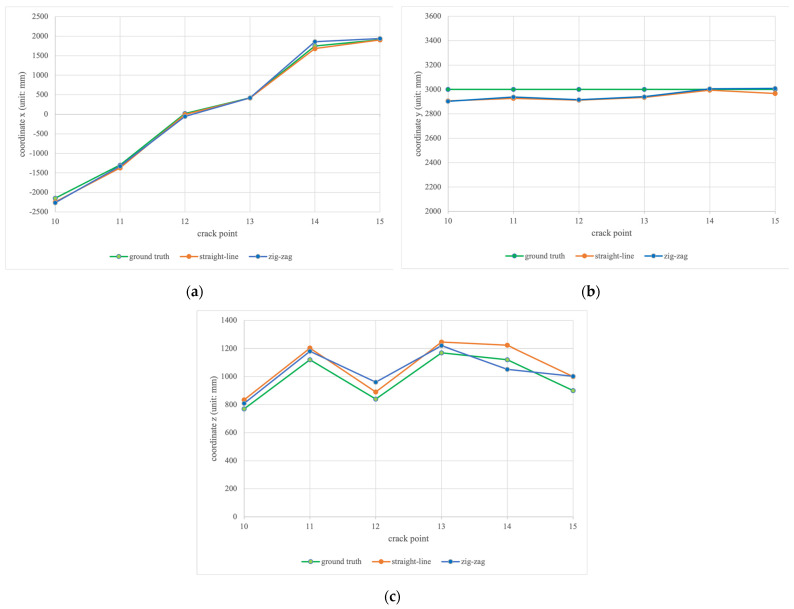
The localization results of (**a**) x coordinates; (**b**) y coordinates; (**c**) z coordinates on the unseen cracks.

To evaluate the generalization ability of improved YOLOv4 model, flight tests on unseen cracks also have been conducted in the laboratory to analyze the crack localization performance on unseen cracks. Likewise, the ground truth positions of the unseen cracks are presented in upper rows, and the localization measurement/estimation values and errors along straight-line and zig-zag trajectories are listed in the middle and bottom rows in Table 7, respectively. Meanwhile, the crack localization results on unseen cracks are visualized in Figure 20. In general, we can observe the measurements of unseen cracks positions follow the ground truth. Particularly, our work achieves the minimum errors of x, y, and z on the unseen cracks are −1, 5, and 39 mm, while the maximum errors of x, y, and z are −116, −97 and 120 mm. Referring to Figure 20, it can be seen that our model demonstrates good generalization performance on unseen cracks.

The Root-Mean-Square errors (RMSE) described in Equation (7) in various flight tests are also employed to evaluate the crack localization performance of the proposed inspection system. Table 8 shows the best results of the RMSE errors of 57 mm, 63 mm, and 79 mm in x, y, and z coordinates, respectively.

(7) $$RMSE=\sqrt{\frac{\sum_{i=1}^{N}{\left({Measured}_{i}-{Ground\;Truth}_{i}\right)}^{2}}{N}}$$

The 3D localization error is computed by Equation (8), *est* denotes the estimation value, and *gt* denotes the ground truth, respectively. The results are presented in Table 9. Likewise, Crack 9 is out of the geo-fence of the VICON tracking system in the laboratory. Thus, the experimental data of Crack 9 are excluded in this section. Overall, the 130 mm average localization error was demonstrated.

(8) $$Distance=\sqrt{{\left(x_{est}-x_{gt}\right)}^{2}+{\left(y_{est}-y_{gt}\right)}^{2}+{\left(z_{est}-z_{gt}\right)}^{2}}$$

## 4. Discussion and Future Work

With drone imagery in Section 3.1, our proposed detector demonstrates its real-time multi-crack detection performance with 90.02% *mAP* on our crack dataset, which outperforms the YOLOv4-original model by 5.23% in terms of *mAP.* We have demonstrated its real-time performance onboard, and it has successfully localized all crack points in the experimental setup. By comparing the ground truth values in Section 3.3, our inspection system has achieved localization accuracy with RMSE errors of 57, 63, and 79 mm in x, y, and z coordinates, respectively, and with a distance error in 3D space of 130 mm. In addition, it has also demonstrated its generalization capability to detect cracks in unseen images in Section 3.2. Furthermore, two different inspection trajectories (i.e., straight-line and zig-zag trajectories) were designed to examine the system performance along inspection paths with different degrees of inspection coverage, and we have verified that the detection and localization performance of the proposed inspection system is independent of the inspection paths (with rather small differences in RMSE for straight-line and zig-zag trajectories).

Generally, we can observe that the localization measurements of the 3D crack positions follow the ground truth in Figure 19 and Figure 20. However, some localization errors are still greater than 100 mm. We conjecture that the depth variance of the RGBD camera is the main source of the errors. Especially at the 4 m inspection range, the depth measurement accuracy of the RGBD camera is around 2%. Furthermore, the error of the generated bounding box from the learning model is another major source in relation to the crack localization accuracy, since the proposed method transforms the coordinate of the 2-D crack center to obtain the coordinate of the 3-D crack positions.

Although the current system is robust in detecting cracks with different concrete textures, it still has a limitation on detecting cracks under extreme illumination conditions, for example, over or under exposures, high brightness, and shades. Because our models are trained with the crack dataset collected under normal lighting conditions, it may not be robust to these challenging conditions. To enhance our model performance, more crack images under different illumination conditions will be added to the training dataset in the future.

This work mainly focuses on enhancing crack detection and localization performance. However, the current localization module does not work well in GPS-denial environments. In the future, the localization module incorporating the simultaneous localization and mapping (SLAM) technique will further improve the localization performance in real applications. Moreover, in order to maximize the inspection coverage for complex structures at a low cost, a global optimal inspection path planning module will be further investigated. Finally, and most importantly, more field tests will be broadly carried out to examine the performance and feasibility of the proposed system.

## 5. Conclusions

In this work, a learning-based real-time autonomous crack inspection system on UAVs incorporating an attention mechanism is proposed. We have labelled a crack dataset that includes 4000 images. Moreover, we have proved its multi-cracks detection performance with 90.02% *mAP* on our customized dataset, which outperforms the original YOLOv4 model with a 5.23% higher *mAP*. In addition, the proposed inspection system works fully onboard and solely utilizes an RGBD camera to precisely locate the crack positions of RMSE errors of 57, 63, and 79 mm in the x, y, and z coordinates, respectively. Moreover, it accurately computed crack positions with a distance error in the 3D world coordinate of 130 mm. Furthermore, we have demonstrated that its localization performance is relatively independent of the inspection trajectory. Overall, the proposed inspection system has achieved cm-level localization accuracy, and various experiment results have verified its robustness and feasibility of performing real-world inspection tasks.

## Figures and Tables

**Table 1:** Specifications of the workstation used to train different detector models.

Item	Description
Operating System	Ubuntu 18.04 (LTS)
CPU	Intel Core i7-10700KF@3.80 GHz
Memory	64 GB
GPU	NVIDIA GeForce RTX3090

**Table 2:** The physical dimensions of cracks in our customized dataset.

Dimension (mm)	Crack 1	Crack 2	Crack 3	Crack 4	Crack5	Crack 6	Crack 7	Crack 8	Crack 9
Width	190	430	310	420	190	230	190	170	140
Height	140	40	200	30	140	130	140	180	90

**Table 3:** The physical dimensions of cracks in the unseen dataset.

Dimension (mm)	Crack 10	Crack 11	Crack 12	Crack 13	Crack 14	Crack 15
Width	50	80	190	140	190	40
Height	150	110	140	110	105	85

**Table 4:** Crack detection performance comparisons on customized datasets.

Models	Precision	Recall	${mAP}_{50}\;*$
YOLOv4-original	0.84	0.81	84.79%
YOLOv4-SE * (our method)	**0.85**	**0.88**	**90.02%**
YOLOv4-tiny-original	0.81	0.84	82.37%
YOLOv4-tiny-SE * (our method)	**0.83**	**0.96**	**85.46%**

* *mAP* denotes mean average precision value; SE denotes Squeeze-and-Excitation.

**Table 5:** Comparative results of the crack detection performance on the UAPD dataset.

Models	Precision	Recall	${mAP}_{50}\;*$
YOLOv4-original	0.67	0.54	45.15%
YOLOv4-SE * (ours)	**0.76**	**0.48**	**48.69%**
YOLOv4-tiny-original	0.57	0.50	43.43%
YOLOv4-tiny-SE * (ours)	**0.62**	**0.55**	**45.02%**

* *mAP* denotes mean average precision value; SE denotes Squeeze-and-Excitation.

**Table 6:** The comparison between ground-truth positions and measured positions on the training cracks.

Measurement (mm)	Crack 1	Crack 2	Crack 3	Crack 4	Crack 5	Crack 6	Crack 7	Crack 8
Ground truth x	−2740	−2100	−1170	−440	140	900	1860	1930
Ground truth y	3000	3000	3000	3000	3000	3000	3000	3000
Ground truth z	430	1030	730	460	780	1130	1080	470
**Straight-line trajectory inspection result**								
Measured x	−2570	−2079	−1190	−476	113	832	1818	1893
Measured y	2984	2931	2932	2980	2900	2903	2933	2935
Measured z	584	1054	720	522	895	1221	1182	590
Error x	170	21	−20	−36	−27	−68	−42	−37
Error y	−16	−69	−68	−20	−100	−97	−67	−65
Error z	154	24	−10	62	115	91	102	120
**Zig-zag trajectory inspection result**								
Measured x	−2868	−2253	−1181	−468	122	853	1841	1871
Measured y	3190	3151	2960	2973	2931	2911	2907	2914
Measured z	570	1253	658	510	875	1187	1166	538
Error x	−128	−153	−11	−28	−18	−47	−19	−59
Error y	190	151	−40	−27	−69	−89	−93	−86
Error z	140	223	−72	50	95	57	86	68

**Table 7:** The comparison between ground truth positions and measured positions on unseen cracks.

Measurement (mm)	Crack 10	Crack 11	Crack 12	Crack 13	Crack 14	Crack 15
Ground truth x	−2150	−1300	20	420	1750	1910
Ground truth y	3000	3000	3000	3000	3000	3000
Ground truth z	770	1120	840	1170	1120	900
**Straight-line trajectory inspection result**						
Measured x	−2243	−1370	−8	415	1681	1904
Measured y	2906	2926	2912	2935	2995	2966
Measured z	834	1203	890	1246	1223	1001
Error x	−93	−70	−28	−5	−69	−6
Error y	−94	−74	−88	−65	−5	−34
Error z	64	83	50	76	103	101
**Zig-zag trajectory inspection result**						
Measured x	−2266	−1326	−57	419	1858	1939
Measured y	2903	2938	2915	2940	3005	3009
Measured z	809	1180	960	1221	1052	1003
Error x	−116	−26	−77	−1	108	29
Error y	−97	−62	−85	−60	5	9
Error z	39	60	120	51	−68	103

**Table 8:** Localization accuracy assessment results in terms of RMSE.

Performance on Training Cracks		Performance on Unseen Cracks	
Straight-Line Trajectory Results			
Coordinate	RMSE Error (Unit: mm)	Coordinate	RMSE Error (Unit: mm)
x	70	x	57
y	69	y	68
z	96	z	82
**Zig-zag trajectory results**			
x	77	x	74
y	106	y	63
z	112	z	79

**Table 9:** Localization accuracy assessment—distance errors in 3D space.

Distance Error (mm)	Crack 1	Crack 2	Crack 3	Crack 4	Crack 5	Crack 6	Crack 7	Crack 8
Straight-line inspection	230	76	72	75	155	149	129	141
Zig-zag inspection	268	310	83	63	119	116	128	124
**Distance error (mm)**	**Crack 10**	**Crack 11**	**Crack 12**	**Crack 13**	**Crack 14**	**Crack 15**	**Average**	
Straight-line inspection	147	132	105	100	125	106	**130**	
Zig-zag inspection	156	91	166	78	128	108		

## Data Availability

The data presented in this study are available in https://github.com/everskyrube/acis (accessed on 21 March 2023).

## Supplementary Materials

The following supporting information is available online at: https://youtu.be/4HIoySNRzHI (accessed on 21 March 2023), Video: Real-time Autonomous Crack Inspection System on UAV.
